# Methods and Evaluation Criteria for Apps and Digital Interventions for Diabetes Self-Management: Systematic Review

**DOI:** 10.2196/18480

**Published:** 2020-07-06

**Authors:** Dillys Larbi, Pietro Randine, Eirik Årsand, Konstantinos Antypas, Meghan Bradway, Elia Gabarron

**Affiliations:** 1 Norwegian Centre for E-health Research University Hospital of North Norway Tromsø Norway; 2 Department of Computer Science Faculty of Science and Technology UiT The Arctic University of Norway Tromsø Norway; 3 SINTEF Digital Oslo Norway; 4 Department of Clinical Medicine Faculty of Health Sciences UiT The Arctic University of Norway Tromsø Norway

**Keywords:** self-management, diabetes mellitus, mobile applications, computer communication networks, mHealth, eHealth, health care evaluation mechanisms

## Abstract

**Background:**

There is growing evidence that apps and digital interventions have a positive impact on diabetes self-management. Standard self-management for patients with diabetes could therefore be supplemented by apps and digital interventions to increase patients’ skills. Several initiatives, models, and frameworks suggest how health apps and digital interventions could be evaluated, but there are few standards for this. And although there are many methods for evaluating apps and digital interventions, a more specific approach might be needed for assessing digital diabetes self-management interventions.

**Objective:**

This review aims to identify which methods and criteria are used to evaluate apps and digital interventions for diabetes self-management, and to describe how patients were involved in these evaluations.

**Methods:**

We searched CINAHL, EMBASE, MEDLINE, and Web of Science for articles published from 2015 that referred to the evaluation of apps and digital interventions for diabetes self-management and involved patients in the evaluation. We then conducted a narrative qualitative synthesis of the findings, structured around the included studies’ quality, methods of evaluation, and evaluation criteria.

**Results:**

Of 1681 articles identified, 31 fulfilled the inclusion criteria. A total of 7 articles were considered of high confidence in the evidence. Apps were the most commonly used platform for diabetes self-management (18/31, 58%), and type 2 diabetes (T2D) was the targeted health condition most studies focused on (12/31, 38%). Questionnaires, interviews, and user-group meetings were the most common methods of evaluation. Furthermore, the most evaluated criteria for apps and digital diabetes self-management interventions were cognitive impact, clinical impact, and usability. Feasibility and security and privacy were not evaluated by studies considered of high confidence in the evidence.

**Conclusions:**

There were few studies with high confidence in the evidence that involved patients in the evaluation of apps and digital interventions for diabetes self-management. Additional evaluation criteria, such as sustainability and interoperability, should be focused on more in future studies to provide a better understanding of the effects and potential of apps and digital interventions for diabetes self-management.

## Introduction

As the number of people with diabetes continues to rise worldwide [1], the need to increase patients’ self-management skills is crucial to improve clinical outcomes and reduce health-related costs [2,3]. There is growing evidence that apps and digital interventions such as websites (web), social media, and other online services have a positive impact on diabetes self-management [4-12], suggesting that standard self-management could be supplemented by digital interventions to aid and improve patients’ skills [4-12]. While some apps and digital interventions have benefited patients, not all of them seem to be based on research, and some of these digital interventions could even compromise the safety of patients with diabetes [13].

To improve diabetes self-management with apps and digital interventions, the World Health Organization and the European Commission [14,15] deem it necessary that the available apps and digital interventions are accurate and reliable. Several initiatives, models, and frameworks suggest how some of these apps and digital interventions could be evaluated [16-19]. These approaches commonly name background information, privacy and security, evidence on the provided information, ease of use, or interoperability as issues that need to be addressed [16-18]. Regarding how to evaluate these criteria, several methods of different complexity have been proposed. These include simple questions to be answered by health care professionals (HCPs) and patients, whereas more complex methodology approaches, such as laboratory-based testing, field testing, and N-of-1 design, are used by researchers [18,20]. Although the aforementioned issues are relevant for diabetes self-management apps and digital interventions, a more specific approach is needed for assessing the growing number and rapidly changing functionalities of these digital diabetes self-management interventions.

Another relevant issue is who should be involved in these evaluations. As patients are often required to make critical decisions based on their own generated health information [21], people with diabetes should be involved in these evaluations. However, a previous assessment of digital health interventions demonstrated limited consideration of user perceptions, and also that of health care personnel [22].

In this systematic review, we identify the specific methods and evaluation criteria that were used to assess apps and digital interventions for diabetes self-management. We also report how patients were involved in these assessments.

## Methods

This review followed the PRISMA approach [23], and its systematic review protocol is registered in PROSPERO (Registration number: CRD42018115246).

### Data Sources and Search Strategy

We performed a single data search in June 2018. The search strategy covered all studies that assessed diabetes self-management apps and digital interventions, involved patients, and were published in English after 2015. We chose a short search period to get a rapid overview of the most recent methods and evaluation criteria. The search strategy covered the following databases: CINAHL, EMBASE, MEDLINE, and Web of Science. The full search strategy is available in Multimedia Appendix 1.

### Inclusion and Exclusion Criteria

We included articles for review if they were (1) primary studies referring to the evaluation of apps or digital interventions for diabetes self-management; and (2) involved patients in the evaluation.

Article were excluded if (1) the evaluation only measured medical values (ie, weight, glycated hemoglobin [HbA_1c_], blood glucose); (2) it was not a primary study; (3) it did not focus on apps or digital interventions for diabetes self-management; (4) the full-text was not available; (5) it was not a peer-reviewed publication; (6) it was not in English; or (7) it was published before 2015.

### Eligibility and Data Collection Procedure

We uploaded all references captured by the search strategy to Rayyan and EndNote and removed duplicates. The eligibility of the articles was assessed in two stages. In the first stage, 2 independent reviewers (PR and EG) examined all titles and abstracts. Eligibility doubts were discussed and agreed with a third and fourth reviewer (KA and EÅ). In the second stage, the full texts of the selected articles were carefully examined by 2 independent reviewers (PR and EG) to confirm their eligibility.

Two reviewers (PR and MB) independently extracted and recorded the data from these articles on an Excel spreadsheet (Microsoft). We extracted the following information from each article: type of platform, targeted health condition, study population, methods of evaluation, and evaluation criteria. Incongruences with the data extraction were discussed among the research group.

### Confidence in the Evidence and Risk of Bias Assessment

Two reviewers (EG and KA) assessed the confidence in the evidence and risk of bias of the articles. We used an approach based on the CERQual guidelines [24] to assess the confidence in the evidence of the qualitative primary studies, by evaluating their methodological limitations, relevance, and adequacy. We followed the GRADE guidelines [25] to assess mixed-methods studies, quantitative studies, and randomized trials.

### Strategy for Data Synthesis

We provide a narrative qualitative synthesis of the findings from the included articles, structured around confidence in the evidence and risk of bias; type of platform (apps, web, or multiplatform [ie, ≥2 types of platform delivering the same intervention in a study]); targeted health condition (type 1 diabetes [T1D], T2D, gestational diabetes mellitus, both T1D and T2D, and unspecified diabetes type); methods of evaluation (questionnaires, interviews, user-group meetings, health measures, system usage analysis, or other); and evaluation criteria (usability, clinical impact, cognitive impact, behavioral impact, feasibility, engagement, acceptability and acceptance, or security and privacy).

## Results

### Identified and Included Studies

**Figure 1:**
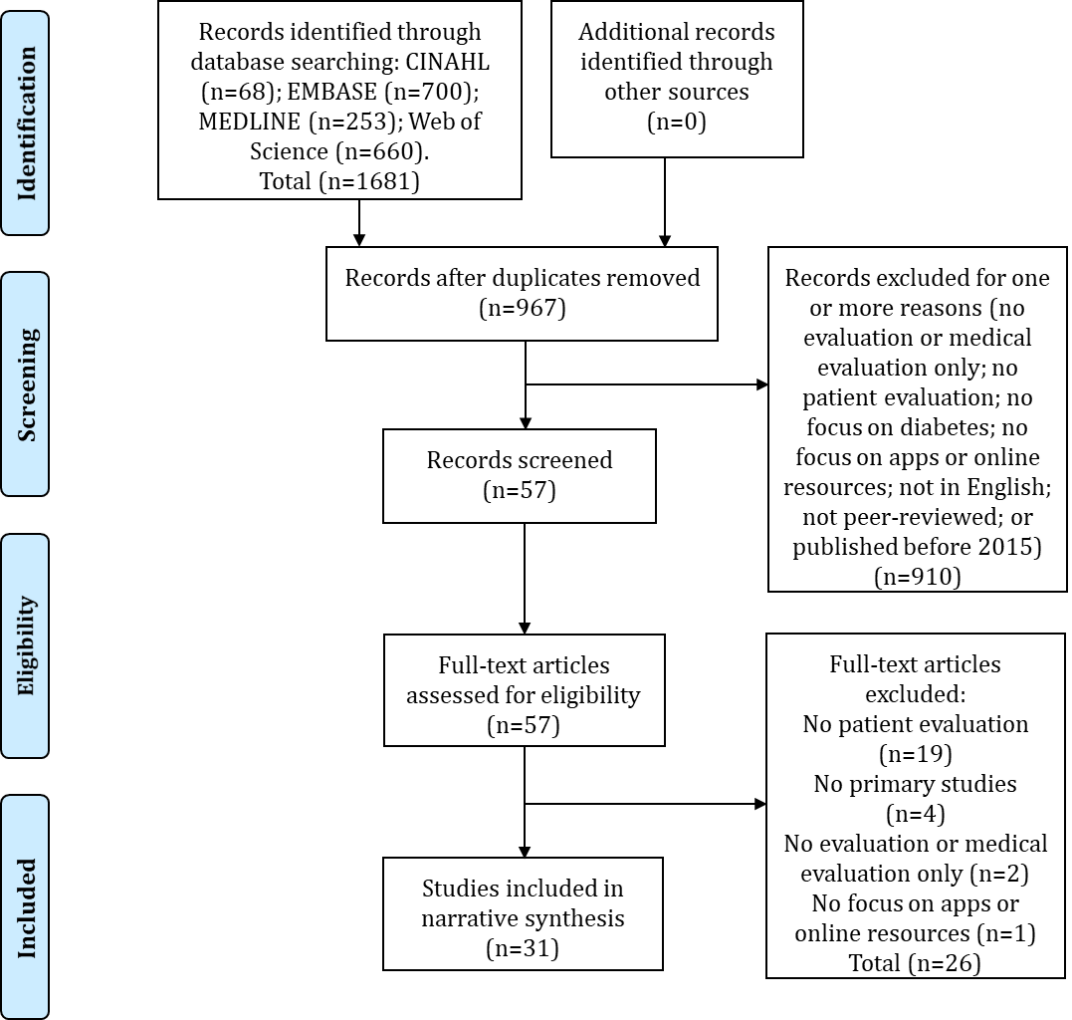
PRISMA flowchart of the selection procedure.

The search strategy resulted in 1681 articles. After removing duplicates, 967 articles remained. In the abstract screening, we excluded 910 articles in accordance with one or more of the exclusion criteria. A total of 57 articles were eligible for full-text screening, 26 of which were excluded (see Multimedia Appendix 2). A total of 31 articles were eventually included in the review [26-56] (see Multimedia Appendix 3). The PRISMA diagram in Figure 1 summarizes the selection process. The confidence in the evidence was considered high in 7 articles [27,33,36,43,51,52,54]; moderate to high in 1 [56]; moderate in 17 [26,28-32,35,37,39,41,42,45,46,48,49,53,55], and low in 6 [34,38,40,44,47,50].

### Study Population

The 31 articles in this review included evaluations from 3689 participants. The number of participants in each study ranged from 7 [41,50] to 1041 [43]. In addition to including patients with diabetes in their evaluations, some of the studies expanded the participant group to include HCPs (8/31, 26%) [26,27,29,37,42,48,53,56], developers (4/31, 13%) [26,44,51,56], researchers (3/31, 10%) [29,38,44], informal caregivers (eg, parents, family members) (4/31, 13%) [29,44,53,56], and others (including experts and other unspecified individuals) (8/31, 26%) [26,29,32,37,38,48,55,56].

### Type of Platform and Targeted Health Condition

**Figure 2:**
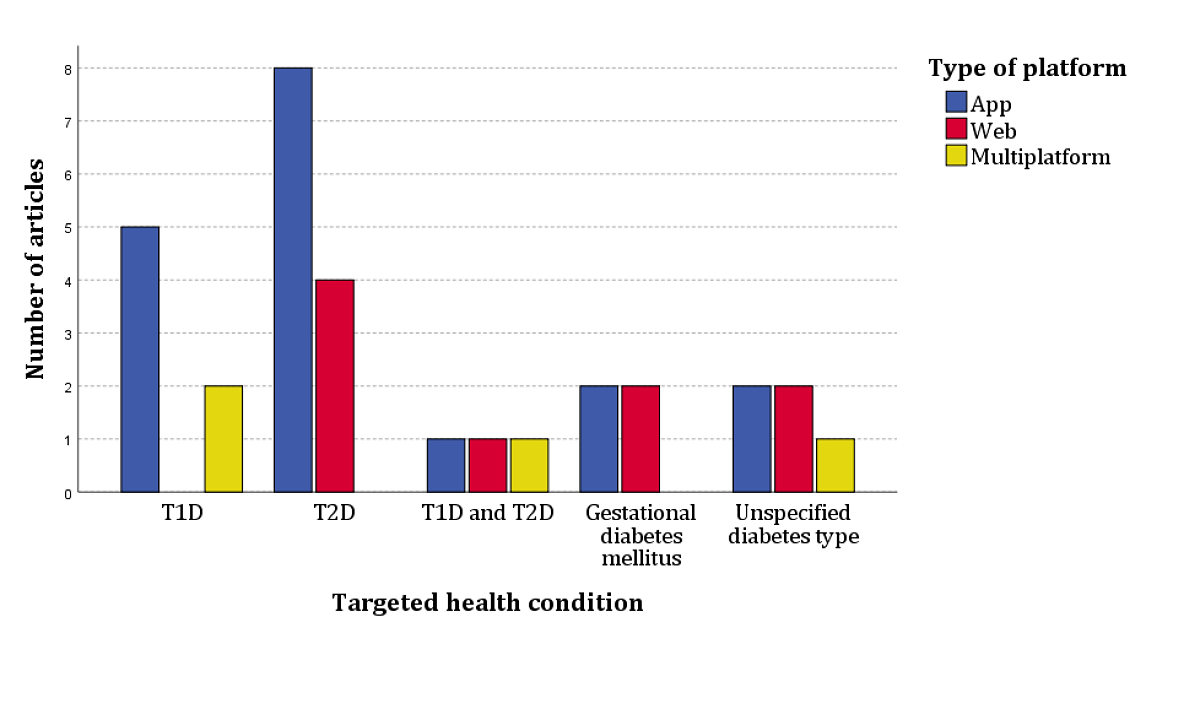
Distribution of types of platform and targeted health conditions among included articles (n=31).

Most of the 31 included studies evaluated interventions delivered via apps (18/31, 58%) [29-34,36-39,41,46-49,52,54,56], followed by web (9/31, 29%) [27,28,35,43,45,50,51,53,55] and multiplatform (4/31, 13%) [26,40,42,44]. In the studies that conducted a randomized controlled trial, the self-management platform was the main mode of intervention compared with a standard paper diary [33], the intervention plus counseling via telephone call [54], and a plain text version of the web intervention [43] as opposed to an interactive version. In addition, the same intervention was referred to by some of the studies: Young with Diabetes app [29,36], My Diabetes My Way [30,55], and WellDoc [31,50]. The evaluated digital self-management interventions targeted mostly T2D (12/31, 38%) [27,31,32,34,43,46-51,54], followed by T1D (7/31, 23%) [29,33,36,40,41,44,56], unspecified diabetes type (5/31, 16%) [26,37,39,53,55], gestational diabetes mellitus (4/31, 13%) [35,38,45,52], and T1D and T2D (3/31, 10%) [28,30,42] (Figure 2).

### Identified Evaluation Methods

The methods of evaluation were grouped into 6 categories: questionnaires, interviews, user-group meetings, health-related measures, system usage analysis, and other measurements. We also identified 20 specific methods that were either used once or multiple times by the studies during the evaluation process.

The interrater agreement for the methods of evaluation was found to be κ=0.550, which represents a moderate agreement [57]. A summary of the specific methods of evaluation and studies that used them is presented in Table 1.

Questionnaires were the most common method used to evaluate diabetes self-management apps and digital interventions (21/31 studies, 68%) [29-40,42,43,45,46,50,51,54-56]. Standardized questionnaires were the most frequently used: 16 in total, each used one or multiple times among 13 studies [31,33-36,38,40,42,43,45,50,51,54]. The second most common method of evaluation was interviews (13/31 studies, 42%) [28,29,31,32,35,36,38,45,48,49,52,53,56], mainly semistructured interviews, which were used 14 times in 11 studies [28,31,32,35,36,45,48,49,52,53,56]. Other methods of evaluation that were identified in the included studies were user-group meetings (11/31, 35%) [26,27,29,37,40-42,45,47,48,56], health-related measures (9/31, 29%) [31,33,36,42,45,49,51,54,55], system usage analysis (8/31, 26%) [29,34,35,42,43,45,51,54], and other measurements (7/31, 23%) [26,37,38,44,48,51,55]. Table 1 summarizes the specific methods of evaluation, the number of times these methods were used, and the number of studies that employed these methods.

Among the 7 studies considered of high confidence in the evidence, the evaluations of the apps and digital diabetes self-management interventions were based mostly on standardized questionnaires [33,36,43,51,54], medical tests [33,36,51,54], and usage log analysis [43,51,54], followed by author-created questionnaires [43,51], semistructured interviews [36,52], focus groups [27], self-reported health measures [33], self-reported usage [43], alpha testing [51], and other oral and written feedback [51].

**Table 1:** Specific methods of evaluation and studies that used them.

Method of evaluation, specific type (n=times used), and details			Reference(s)
**Questionnaires**			
	**Standardized questionnaires (n=26)**		
		Block Food Frequency Assessment	[45]
		Dietary Knowledge, Attitude, and Behavior Questionnaire	[51]
		Health Care Climate Questionnaire	[36]
		Paffenbarger Questionnaire	[45]
		Patient Enablement Instrument	[43]
		Patient Health Questionnaire-9	[50]
		Patient Reported Diabetes Symptoms Scale	[50]
		Perceived Competence in Diabetes	[36]
		Problem Areas in Diabetes-5	[31]
		Problem Areas in Diabetes	[33,36,40,42]
		RAND 36-Item Health Survey 1.0	[33]
		Self-Efficacy for Diabetes Scale	[50]
		System Usability Scale	[33-35,38,40,42]
		The Health Education Impact Questionnaire	[54]
		The Service User Technology Acceptability Questionnaire	[54]
		36-Item Short Form Survey	[50]
	**Author-created questionnaires (n=20)**		
		N/A^a^	[29,30,32,37-39,42,43,45,46,51,55,56]
**User-group meetings**			
	**Focus groups (n=9)**		
		N/A	[27,37,40-42,45,47]
	**Workshops (n=7)**		
		N/A	[26,29,48,56]
**Interviews**			
	**Semistructured interviews (n=14)**		
		N/A	[28,31,32,35,36,45,48,49,52,53,56]
	**Unspecified interview format (n=3)**		
		N/A	[29,38]
**System usage analysis**			
	**Usage log analysis (n=8)**		
		N/A	[34,35,43,45,51,54]
	**Self-reported usage (n=2)**		
		N/A	[42,43]
	**Think-aloud protocol (n=1)**		
		N/A	[29]
**Health-related measures**			
	**Medical tests (n=8)**		
		HbA_1c_	[31,33,36,45,49,51,54,55]
		Fasting blood glucose	[51]
		Blood pressure and cholesterol	[55]
		Gestational weight gain	[45]
	**Self-reported health measures (n=5)**		
		Self-reported blood glucose	[33,42]
		Self-reported physical activity and nutritional habits	[42]
**Other measurements**			
	**Security assessment (n=1)**		
		N/A	[44]
	**Scenarios (n=2)**		
		N/A	[37,38]
	**Cost-effectiveness (n=1)**		
		N/A	[55]
	**Alpha testing (n=1)**		
		N/A	[51]
	**Observation (n=2)**		
		N/A	[48]
	**Rating system (n=1)**		
		Star rating	[26]
	**Heuristics method (n=1)**		
		Bertini’s mobile tool	[37]
	**Anecdotal feedback (n=1)**		
		Open text review	[26]
	**Other oral and written feedback (n=2)**		
		N/A	[44,51]

^a^N/A: not applicable.

**Identified Evaluation Criteria**

The evaluated criteria were grouped into 8 categories: usability, clinical impact, cognitive impact, behavioral impact, feasibility, engagement, acceptability and acceptance, and security and privacy. The included studies evaluated one or several of these identified criteria. The interrater agreement (κ) for the evaluation criteria was found to be 0.563, which represents a moderate agreement [57].

Among the 7 studies considered of high confidence in the evidence, the most commonly evaluated criteria were clinical impact [33,36,51,54], cognitive impact [33,36,43,54], and engagement [43,51,54], followed by usability [33,51], behavioral impact [33,51], and acceptability and acceptance [51,54]. None of these studies considered of high confidence evaluated feasibility or security and privacy.

Qualitative and mixed-method studies that used thematic analysis in their evaluation focused mostly on usability as an evaluation criterion. Three of the studies considered of high confidence in evidence were qualitative and mixed-method studies. Of these, 2 evaluated cognitive impact [36,52] and usability [27,52], and 1 evaluated engagement [36].

**Figure 3:**
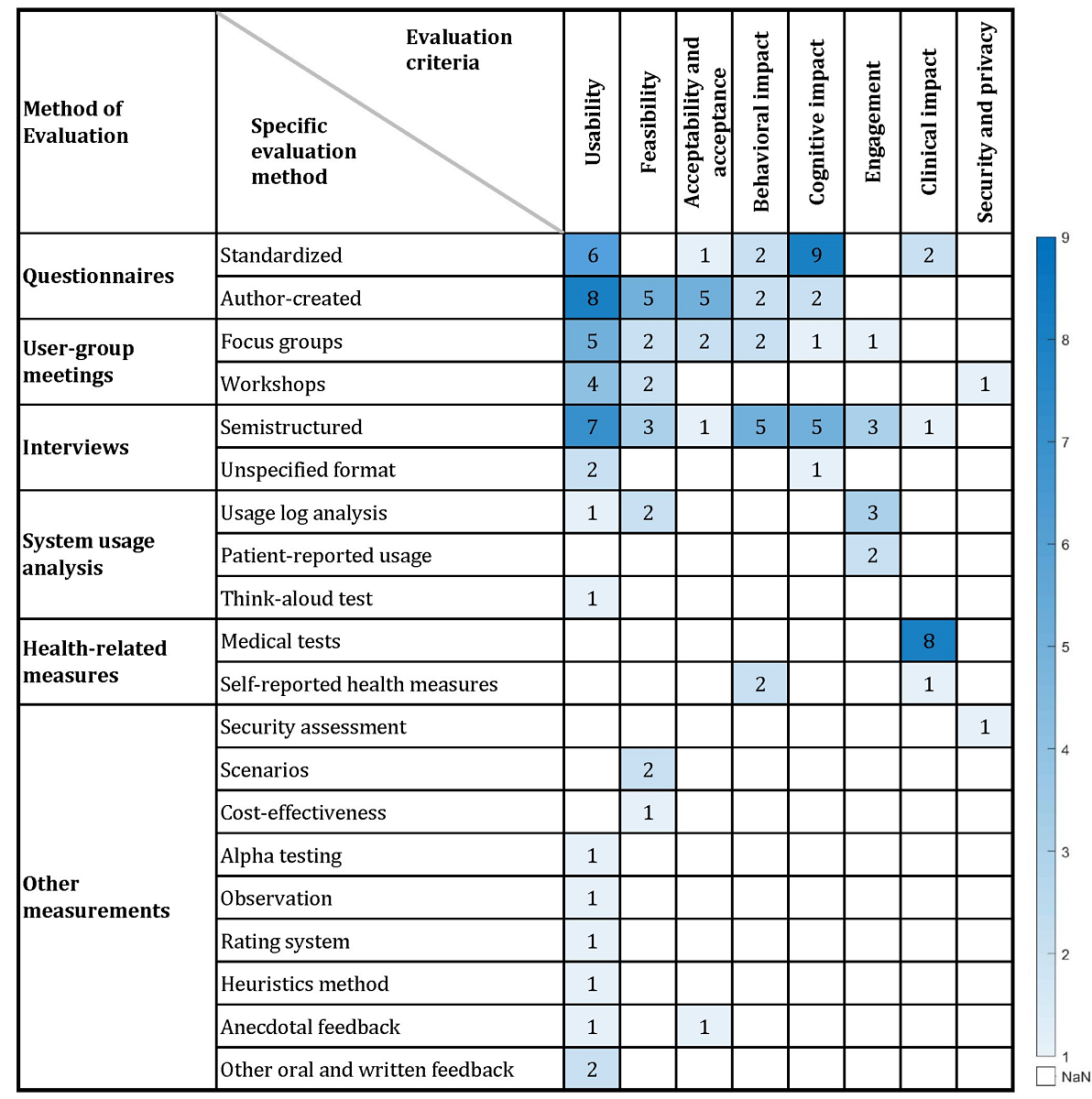
Number of studies using the various methods of evaluation and evaluation criteria. Blank boxes (NaN): No studies within this category.

Figure 3 shows the number of studies that used each of the specific methods to evaluate the identified criteria. It illustrates that several methods were used to evaluate one criterion in a single study. Likewise, some studies evaluated several criteria using one or more of the identified methods of evaluation. For example, of the 31 included studies, 9 [31,33,36,40,42,43,45,50,54] evaluated cognitive impact using standardized questionnaires.

## Discussion

### Summary of the Findings

This review aimed to identify the existing methods and criteria used to assess apps and digital diabetes self-management interventions that involved patients in their evaluations. A total of 31 articles were included in the review, 7 of which were considered of high confidence in the evidence [27,33,36,43,51,52,54]. More than half of the studies (18/31, 58%) focused on the evaluation of apps for diabetes self-management, and 12 of the 31 studies addressed T2D. The most commonly used methods of evaluation were questionnaires, interviews, and user-group meetings. The most used evaluation criteria to assess apps and digital interventions for diabetes self-management were cognitive impact, clinical impact, and usability.

### Specific Evaluation Criteria and Diabetes Patients’ Assessment

In our review, we have found that studies dealing specifically with apps and digital interventions for diabetes self-management focus on the evaluation of more technology-related and users’ interaction aspects (ie, acceptability and acceptance, and engagement). In addition, these studies focus on the impact that these digital self-management interventions have on the individual. Behavioral impact, cognitive impact, and clinical impact were used as relevant criteria for assessing all types of digital interventions for diabetes self-management. It is vital to measure the interventions’ impact on their users because those that have shown benefits related to behavioral, cognitive, and clinical impact could reduce health-related costs [2,3].

Evidence shows that involving individuals in the assessment of different health interventions has a positive impact on health [58]. We found few articles (n=31) in this review that involved patients in the evaluation of apps and digital interventions for diabetes self-management. The evaluations in which patients were involved in mostly focused on usability and cognitive impact. Evaluation criteria that could measure patients’ continuous use of these apps and digital interventions for self-management could supplement both their qualitative responses and the more static traditional and clinical criteria. This is an opportunity for improvement, as none of the studies in this review evaluated the same criterion using both qualitative results from patients and quantitative measures.

Involving patients with diabetes in assessing apps and digital self-management interventions, and obtaining their feedback regarding additional evaluation criteria could also increase our knowledge about the features that support engagement with these technologies. This could also help create better digital health interventions that encourage more continuous and effective use [59]. The most common methods of evaluation with the patients were questionnaires, interviews, and user-group meetings. Simple methods such as these elicit the opinion and perceptions of users, as well as encourage them to critically analyze self-management apps and digital interventions. Therefore, such methods should be used in conjunction with complex methods used by researchers and developers [18,20], especially to measure the same criterion.

### Improving Reported Evaluations of Digital Interventions for Diabetes Self-Management

Apps and digital health interventions have evolved quickly. Yet, compared with other sectors, the health industry seems to be behind with regard to digitalization [60]. Currently, most apps and digital interventions for self-management are not recommended as part of the treatment plan, maybe because their design and development do not take into consideration sustainability [61]. In fact, digital health interventions rarely advance beyond a pilot phase [62,63], or the duration of an intervention study.

In 2016, the mobile health (mHealth) evidence reporting and assessment checklist was developed by the World Health Organization to help with reporting evidence of the effectiveness of mHealth interventions [64]. The checklist recommended reporting on items that touch on sustainability, scalability, and transparency, such as infrastructure, interoperability, contextual adaptability, and replicability, which we still see are not much focused on in today’s studies. Future studies should also consider these evaluation criteria in addition to gender and equity issues associated with the use of apps and digital interventions for diabetes self-management.

Evaluation reports for apps and digital interventions for diabetes self-management must be standardized, as recommended by the CONSORT-EHEALTH guidelines for reporting digital health interventions [65]. The lack of standardization made it challenging to compare studies as different authors used different terminologies to describe the same evaluation criterion. For example, one study [37] used the term *heuristics evaluation*, which was grouped under usability because it evaluated measures such as the visibility of app status, ease of input, and readability. Likewise, another study [32] evaluated *satisfaction*, which falls under usability because it evaluated among others, visual attractiveness and ease of use.

As electronic health (eHealth) research is a multidisciplinary field, we assume that the authors chose these terms based on the various educational or professional backgrounds and the corresponding target audiences. By following the World Health Organization classification of digital health interventions [66], terminologies related to the evaluation of apps and digital interventions for diabetes self-management could be standardized to facilitate straightforward interpretation and aggregation of research evidence.

### Association Between Methods Used and Criteria Evaluated

In our review we have found that there was an almost even split of studies that used standardized questionnaires, author-created questionnaires, and semistructured interviews to evaluate usability. Our results are to some extent in line with the findings of a previous review that found that usability was mainly assessed though polls and questionnaires [67]. The usability of a digital self-management intervention is crucial to its successful adoption, its acceptance, and the individual’s engagement with it. In addition, we found that cognitive impact was often assessed not only through standardized questionnaires, but also through semistructured interviews.

Comparing the methods for the evaluation of usability with those for the evaluation of cognitive impact, we identified that it was more common to use author-created questionnaire for usability. A possible explanation might be the wide variety of intervention delivery platforms (eg, different types of apps and online resources) that might create different evaluation needs not captured in existing standardized usability questionnaires. Another explanation might be the different research traditions in different disciplines. Usability might be more often a concern of computer science researchers, whereas cognitive impact a concern of health researchers and professionals.

Finally, health outcomes were almost exclusively evaluated by medical tests, showing the preference of health researchers and professionals in using standardized tests to determine the impact of digital interventions. Several other methods can be used to evaluate multiple criteria; however, depending on the aim and the type of study, researchers must endeavor to exhaust all available methods to ensure consistency of results.

### Feasibility of Using Digital Self-Management Interventions in Clinical Workflow

Although most apps and digital health interventions are intended for self-management, some of them also provide access to the health care system, such as communication with HCPs and electronic health journals. The reviewed studies consistently reported that this is in response to patients’ interest in being able to contact their HCPs or share results (eg, their blood glucose results with their health care team). This was the case not only within our review [35,36,42,44,47,48,56] but also by industry research groups [68,69]. This implies the potential and expectation for further involvement of HCPs in patients’ use of apps and digital interventions for diabetes self-management.

Several studies, including many in this review, have shown that involving HCPs in digital interventions is associated with improved self-management of diabetes and the success of these interventions [31,48,49,52,70-72]. Therefore, studies focusing on apps and digital interventions for diabetes self-management should evaluate the possibilities of effortlessly integrating these interventions in the workflow of HCPs—the connection and interaction with electronic health journals and other existing health information systems. Such an integration can be achieved by evaluating the infrastructure needed for digital self-management interventions [64].

### Limitations and Strengths

The search for articles covered a short period (2015-2018) and focused on articles published in the English language. Therefore, we may have missed relevant studies that reported additional evaluation methods or evaluation criteria. Our interrater agreement of the data extraction was only moderate; however, all incongruences were discussed among the research group. Our findings have provided a useful overview of the recent evaluation methods and criteria that researchers are using to assess current apps and digital interventions for diabetes self-management. Furthermore, our review included both quantitative and qualitative studies which provided a better characterization of different evaluation methods and criteria that are being used to assess digital diabetes self-management interventions.

### Conclusions

There are only few studies that involved patients in the evaluation of apps and digital interventions for diabetes self-management, and even fewer still considered of high confidence in the evidence. The most common evaluation methods were questionnaires, interviews, and user-group meetings, whereas evaluation criteria were cognitive impact, clinical impact, and usability. Studies with high confidence in the evidence did not evaluate feasibility or security and privacy, neither were patients involved in evaluating the latter criterion which was evaluated in only 2 [29,44] of the included studies.

It is important to the successful implementation and continuous use of apps and digital interventions for diabetes self-management that patients are involved in evaluating every criteria. In that way, they can contribute to the development and modification of these digital interventions to better meet their specific self-management needs. Furthermore, the methods and criteria evaluated in digital diabetes self-management interventions should be expanded to assess and ensure sustainability and interoperability. In addition, studies should evaluate the association between cognitive, clinical, and behavioral impact of these apps and digital interventions, and health-related costs for individuals with diabetes. This could help improve health care associated with the management of diabetes and promote the incorporation of apps and digital interventions for self-management in the services provided at health care facilities.

## Appendix

Multimedia Appendix 1 Search strategy (search date: June 21, 2018).

Multimedia Appendix 2 List of rejected articles after full-text review (n=26).

Multimedia Appendix 3 Articles included in qualitative synthesis (n=31).

Multimedia Appendix 4 PRISMA checklist.

## Abbreviations

HbA_1c_: glycated hemoglobin
HCPs: health care professionals
T1D: type 1 diabetes
T2D: type 2 diabetes

## References

[1] World Health Organization (2019). Diabetes.

[2] Barker I, Steventon A, Williamson R, Deeny SR (2018). Self-management capability in patients with long-term conditions is associated with reduced healthcare utilisation across a whole health economy: cross-sectional analysis of electronic health records. BMJ Qual Saf.

[3] Grady PA, Gough LL (2014). Self-management: a comprehensive approach to management of chronic conditions. Am J Public Health.

[4] Wilhide ICC, Peeples MM, Kouyaté RCA (2016). Evidence-Based mHealth Chronic Disease Mobile App Intervention Design: Development of a Framework. JMIR Res Protoc.

[5] Marcolino MS, Oliveira JAQ, D'Agostino M, Ribeiro AL, Alkmim MBM, Novillo-Ortiz D (2018). The Impact of mHealth Interventions: Systematic Review of Systematic Reviews. JMIR Mhealth Uhealth.

[6] Cotter AP, Durant N, Agne AA, Cherrington AL (2014). Internet interventions to support lifestyle modification for diabetes management: a systematic review of the evidence. J Diabetes Complications.

[7] Gabarron E, Årsand E, Wynn R (2018). Social Media Use in Interventions for Diabetes: Rapid Evidence-Based Review. J Med Internet Res.

[8] Hou C, Carter B, Hewitt J, Francisa T, Mayor S (2016). Do Mobile Phone Applications Improve Glycemic Control (HbA1c) in the Self-management of Diabetes? A Systematic Review, Meta-analysis, and GRADE of 14 Randomized Trials. Diabetes Care.

[9] Ramadas A, Quek KF, Chan CKY, Oldenburg B (2011). Web-based interventions for the management of type 2 diabetes mellitus: a systematic review of recent evidence. Int J Med Inform.

[10] Shan R, Sarkar S, Martin SS (2019). Digital health technology and mobile devices for the management of diabetes mellitus: state of the art. Diabetologia.

[11] Greenwood DA, Gee PM, Fatkin KJ, Peeples M (2017). A Systematic Review of Reviews Evaluating Technology-Enabled Diabetes Self-Management Education and Support. J Diabetes Sci Technol.

[12] Huang Z, Soljak M, Boehm BO, Car J (2018). Clinical relevance of smartphone apps for diabetes management: A global overview. Diabetes Metab Res Rev.

[13] Huckvale K, Adomaviciute S, Prieto JT, Leow MK, Car J (2015). Smartphone apps for calculating insulin dose: a systematic assessment. BMC Med.

[14] European Commission (2017). Report of the Working Group on mHealth Assessment Guidelines.

[15] World Health Organization (2011). mHealth. New Horizons for Health Through Mobile Technologies.

[16] Henson P, David G, Albright K, Torous J (2019). Deriving a practical framework for the evaluation of health apps. Lancet Digital Health.

[17] Health on the net (2019). HON Code.

[18] American Psychiatric Association (2020). App Evaluation Model.

[19] National Institute for Health and Care Excellence (2019). Evidence Standards Framework for Digital Health Technologies.

[20] Jake-Schoffman DE, Silfee VJ, Waring ME, Boudreaux ED, Sadasivam RS, Mullen SP, Carey JL, Hayes RB, Ding EY, Bennett GG, Pagoto SL (2017). Methods for Evaluating the Content, Usability, and Efficacy of Commercial Mobile Health Apps. JMIR Mhealth Uhealth.

[21] Lewis TL, Wyatt JC (2014). mHealth and mobile medical Apps: a framework to assess risk and promote safer use. J Med Internet Res.

[22] Bradway M, Carrion C, Vallespin B, Saadatfard O, Puigdomènech E, Espallargues M, Kotzeva A (2017). mHealth Assessment: Conceptualization of a Global Framework. JMIR Mhealth Uhealth.

[23] Moher D, Liberati A, Tetzlaff J, Altman DG (2009). Preferred reporting items for systematic reviews and meta-analyses: the PRISMA statement. Ann Intern Med.

[24] Lewin S, Bohren M, Rashidian A, Munthe-Kaas H, Glenton C, Colvin CJ, Garside R, Noyes J, Booth A, Tunçalp O, Wainwright M, Flottorp S, Tucker JD, Carlsen B (2018). Applying GRADE-CERQual to qualitative evidence synthesis findings-paper 2: how to make an overall CERQual assessment of confidence and create a Summary of Qualitative Findings table. Implement Sci.

[25] Balshem H, Helfand M, Schünemann HJ, Oxman AD, Kunz R, Brozek J, Vist GE, Falck-Ytter Y, Meerpohl J, Norris S, Guyatt GH (2011). GRADE guidelines: 3. Rating the quality of evidence. J Clin Epidemiol.

[26] Ashurst EJ, Jones RB (2017). Is the Health App Challenge approach of patient-led application conception, development, and review worthwhile?. Health Policy Technol.

[27] Bernhard G, Mahler C, Seidling HM, Stützle M, Ose D, Baudendistel I, Wensing M, Szecsenyi J (2018). Developing a Shared Patient-Centered, Web-Based Medication Platform for Type 2 Diabetes Patients and Their Health Care Providers: Qualitative Study on User Requirements. J Med Internet Res.

[28] Brady E, Segar J, Sanders C (2017). Accessing support and empowerment online: The experiences of individuals with diabetes. Health Expect.

[29] Castensøe-Seidenfaden P, Husted GR, Teilmann G, Hommel E, Olsen BS, Kensing F (2017). Designing a Self-Management App for Young People With Type 1 Diabetes: Methodological Challenges, Experiences, and Recommendations. JMIR Mhealth Uhealth.

[30] Conway N, Campbell I, Forbes P, Cunningham S, Wake D (2016). mHealth applications for diabetes: User preference and implications for app development. Health Informatics J.

[31] Desveaux L, Shaw J, Saragosa M, Soobiah C, Marani H, Hensel J, Agarwal P, Onabajo N, Bhatia RS, Jeffs L (2018). A Mobile App to Improve Self-Management of Individuals With Type 2 Diabetes: Qualitative Realist Evaluation. J Med Internet Res.

[32] Dewi DS, Irfoni AR, Rahman A (2017). Kansei Engineering Approach for Designing a Self-monitoring Blood Glucose Application. IJTech.

[33] Drion I, Pameijer LR, van Dijk PR, Groenier KH, Kleefstra N, Bilo HJG (2015). The Effects of a Mobile Phone Application on Quality of Life in Patients With Type 1 Diabetes Mellitus: A Randomized Controlled Trial. J Diabetes Sci Technol.

[34] Georgsson M, Staggers N (2016). Quantifying usability: an evaluation of a diabetes mHealth system on effectiveness, efficiency, and satisfaction metrics with associated user characteristics. J Am Med Inform Assoc.

[35] Gianfrancesco C, Darwin Z, McGowan L, Smith DM, Haddrill R, Carter M, Scott EM, Alwan NA, Morris MA, Albar SA, Cade JE (2018). Exploring the Feasibility of Use of An Online Dietary Assessment Tool (myfood24) in Women with Gestational Diabetes. Nutrients.

[36] Husted GR, Weis J, Teilmann G, Castensøe-Seidenfaden P (2018). Exploring the Influence of a Smartphone App (Young with Diabetes) on Young People's Self-Management: Qualitative Study. JMIR Mhealth Uhealth.

[37] Jeon E, Park H (2018). Development of the IMB Model and an Evidence-Based Diabetes Self-management Mobile Application. Healthc Inform Res.

[38] Jo S, Park H (2016). Development and Evaluation of a Smartphone Application for Managing Gestational Diabetes Mellitus. Healthc Inform Res.

[39] Kim YJ, Rhee SY, Byun JK, Park SY, Hong SM, Chin SO, Chon S, Oh S, Woo J, Kim SW, Kim YS (2015). A Smartphone Application Significantly Improved Diabetes Self-Care Activities with High User Satisfaction. Diabetes Metab J.

[40] Klaassen R, Bul KCM, Op den Akker R, van der Burg GJ, Kato PM, Di Bitonto P (2018). Design and Evaluation of a Pervasive Coaching and Gamification Platform for Young Diabetes Patients. Sensors (Basel).

[41] Knight BA, McIntyre HD, Hickman IJ, Noud M (2016). Qualitative assessment of user experiences of a novel smart phone application designed to support flexible intensive insulin therapy in type 1 diabetes. BMC Med Inform Decis Mak.

[42] Lamprinos I, Demski H, Mantwill S, Kabak Y, Hildebrand C, Ploessnig M (2016). Modular ICT-based patient empowerment framework for self-management of diabetes: Design perspectives and validation results. Int J Med Inform.

[43] Muller I, Rowsell A, Stuart B, Hayter V, Little P, Ganahl K, Müller G, Doyle G, Chang P, Lyles CR, Nutbeam D, Yardley L (2017). Effects on Engagement and Health Literacy Outcomes of Web-Based Materials Promoting Physical Activity in People With Diabetes: An International Randomized Trial. J Med Internet Res.

[44] Neinstein A, Wong J, Look H, Arbiter B, Quirk K, McCanne S, Sun Y, Blum M, Adi S (2016). A case study in open source innovation: developing the Tidepool Platform for interoperability in type 1 diabetes management. J Am Med Inform Assoc.

[45] Nicholson WK, Beckham AJ, Hatley K, Diamond M, Johnson L, Green SL, Tate D (2016). The Gestational Diabetes Management System (GooDMomS): development, feasibility and lessons learned from a patient-informed, web-based pregnancy and postpartum lifestyle intervention. BMC Pregnancy Childbirth.

[46] Park S, Burford S, Nolan C, Hanlen L (2016). The Role of Digital Engagement in the Self-Management of Type 2 Diabetes. Health Commun.

[47] Peng W, Yuan S, Holtz BE (2016). Exploring the Challenges and Opportunities of Health Mobile Apps for Individuals with Type 2 Diabetes Living in Rural Communities. Telemed J E Health.

[48] Petersen M, Hempler NF (2017). Development and testing of a mobile application to support diabetes self-management for people with newly diagnosed type 2 diabetes: a design thinking case study. BMC Med Inform Decis Mak.

[49] Pludwinski S, Ahmad F, Wayne N, Ritvo P (2015). Participant experiences in a smartphone-based health coaching intervention for type 2 diabetes: A qualitative inquiry. J Telemed Telecare.

[50] Quinn CC, Khokhar B, Weed K, Barr E, Gruber-Baldini AL (2015). Older Adult Self-Efficacy Study of Mobile Phone Diabetes Management. Diabetes Technol Ther.

[51] Ramadas A, Chan CKY, Oldenburg B, Hussien Z, Quek KF (2015). A web-based dietary intervention for people with type 2 diabetes: development, implementation, and evaluation. Int J Behav Med.

[52] Skar JB, Garnweidner-Holme LM, Lukasse M, Terragni L (2018). Women's experiences with using a smartphone app (the Pregnant+ app) to manage gestational diabetes mellitus in a randomised controlled trial. Midwifery.

[53] Tieu L, Sarkar U, Schillinger D, Ralston JD, Ratanawongsa N, Pasick R, Lyles CR (2015). Barriers and Facilitators to Online Portal Use Among Patients and Caregivers in a Safety Net Health Care System: A Qualitative Study. J Med Internet Res.

[54] Torbjørnsen A, Småstuen MC, Jenum AK, Årsand E, Ribu L (2018). Acceptability of an mHealth App Intervention for Persons With Type 2 Diabetes and its Associations With Initial Self-Management: Randomized Controlled Trial. JMIR Mhealth Uhealth.

[55] Wake DJ, He J, Czesak AM, Mughal F, Cunningham SG (2016). MyDiabetesMyWay: An Evolving National Data Driven Diabetes Self-Management Platform. J Diabetes Sci Technol.

[56] Zhang Y, Li X, Luo S, Liu C, Liu F, Zhou Z (2018). Exploration of Users' Perspectives and Needs and Design of a Type 1 Diabetes Management Mobile App: Mixed-Methods Study. JMIR Mhealth Uhealth.

[57] Landis JR, Koch GG (1977). The measurement of observer agreement for categorical data. Biometrics.

[58] Campbell M, Escobar O, Fenton C, Craig P (2018). The impact of participatory budgeting on health and wellbeing: a scoping review of evaluations. BMC Public Health.

[59] Adu MD, Malabu UH, Callander EJ, Malau-Aduli AE, Malau-Aduli BS (2018). Considerations for the Development of Mobile Phone Apps to Support Diabetes Self-Management: Systematic Review. JMIR Mhealth Uhealth.

[60] Azzopardi-Muscat N, Ricciardi W, Odone A, Buttigieg S, Zeegers Paget D (2019). Digitalization: potentials and pitfalls from a public health perspective. Eur J Public Health.

[61] Wali S, Keshavjee K, Demers C, Heston TF (2018). Moving towards sustainable electronic health applications. eHealth - Making Health Care Smarter.

[62] Huang F, Blaschke S, Lucas H (2017). Beyond pilotitis: taking digital health interventions to the national level in China and Uganda. Global Health.

[63] Wilson K, Gertz B, Arenth B, Salisbury N (2014). The Journey to Scale: Moving Together Past Digital Health Pilots.

[64] Agarwal S, LeFevre AE, Lee J, L'Engle K, Mehl G, Sinha C, Labrique A (2016). Guidelines for reporting of health interventions using mobile phones: mobile health (mHealth) evidence reporting and assessment (mERA) checklist. BMJ.

[65] Eysenbach G, CONSORT-EHEALTH Group E (2011). CONSORT-EHEALTH: improving and standardizing evaluation reports of Web-based and mobile health interventions. J Med Internet Res.

[66] World Health Organization (2018). Classification of Digital Health Interventions (WHO/RHR/18.06).

[67] Vera F, Noël R, Taramasco C (2019). Standards, Processes and Instruments for Assessing Usability of Health Mobile Apps: A Systematic Literature Review. Stud Health Technol Inform.

[68] Research2Guidance (2018). mHealth Developer Economics: Connectivity in Digital Health.

[69] Research2Guidance (2017). mHealth App Economics 2017: Current Status and Future Trends in Mobile Health.

[70] Muralidharan S, Ranjani H, Anjana RM, Allender S, Mohan V (2017). Mobile Health Technology in the Prevention and Management of Type 2 Diabetes. Indian J Endocrinol Metab.

[71] Holmen H, Torbjørnsen A, Wahl AK, Jenum AK, Småstuen MC, Arsand E, Ribu L (2014). A Mobile Health Intervention for Self-Management and Lifestyle Change for Persons With Type 2 Diabetes, Part 2: One-Year Results From the Norwegian Randomized Controlled Trial RENEWING HEALTH. JMIR Mhealth Uhealth.

[72] Triantafyllidis A, Kondylakis H, Votis K, Tzovaras D, Maglaveras N, Rahimi K (2019). Features, outcomes, and challenges in mobile health interventions for patients living with chronic diseases: A review of systematic reviews. Int J Med Inform.

