# Supplementary material for: Methods and Evaluation Criteria for Apps and Digital Interventions for Diabetes Self-Management: Systematic Review
Source: J Med Internet Res. 2020 Jul 6;22(7):e18480. doi: 10.2196/18480 (PMC7381260; doi:10.2196/18480)
Supplement: Multimedia Appendix 1 [file jmir_v22i7e18480_app1.doc]

**Multimedia Appendix 1**

Search strategy (search date: June 21, 2018)

| **Search strategy** | **Output** |
| --- | --- |
| ”Mobile health” OR ”mHealth” OR ”eHealth” OR “digital health” OR “health app” OR ”app” OR “mobile application” OR ”smartphone” OR ”wearable” OR ”sensor” OR “mobile phone” OR “cell phone” OR ”tablet” OR ”web-based” OR ”online” OR ”internet” OR “Social media” OR ”Facebook” OR ”Twitter” OR “web-sites”  AND ”Self-assessment” OR ”self-management” OR ”self-manage* OR ”self-monitor* OR “Self-care” AND “Assessment“ OR “validation“ OR “evaluation“ OR “toolkit“ OR “framework“ OR “model“ OR “guideline“ OR “recommendations“ OR “guide“ OR “survey“ OR “questionnaire“ OR “checklist“ OR “instrument“ OR “Criteria“ | CINAHL (n=68)  EMBASE (n=700)  MEDLINE (n= 253)  Web of Science (n= 660) |
| **TOTAL** | **1681 (including duplicates)** |
