# Supplementary material for: Methods and Evaluation Criteria for Apps and Digital Interventions for Diabetes Self-Management: Systematic Review
Source: J Med Internet Res. 2020 Jul 6;22(7):e18480. doi: 10.2196/18480 (PMC7381260; doi:10.2196/18480)
Supplement: Multimedia Appendix 2 [file jmir_v22i7e18480_app2.doc]

**Multimedia Appendix 2**

List of rejected articles after full-text review (n= 26)

| **NO PATIENT EVALUATION (n=19)** |
| --- |
| Adu MD, Malabu UH, Callander EJ, Malau-Aduli AEO, Malau-Aduli BS. Considerations for the Development of Mobile Phone Apps to Support Diabetes Self-Management: Systematic Review. JMIR Mhealth Uhealth. 2018 Jun 21;6(6):e10115. PMID:29929949 |
| Alvarado-Martel D, Canas F, Velasco R, Alcubierre N, Lopez-Rios L, Rius F, Novoa FJ, Carrillo A, Hernandez M, Wagner AM, Mauricio D. Design, construction, and implementation of an online platform for patients with type 1 diabetes: EncoDiab. Patient Prefer Adherence. 2015;9:767-75. PMID:605172737 |
| Basilico A, Marceglia S, Bonacina S, Pinciroli F. Advising patients on selecting trustful apps for diabetes self-care. Comput Biol Med. 2016;71:86-96. PMID:26897071 |
| Brzan PP, Rotman E, Pajnkihar M, Klanjsek P. Mobile Applications for Control and Self Management of Diabetes: A Systematic Review. J Med Syst. 2016;40(9):210. PMID:27520615 |
| de Ridder M, Kim J, Jing Y, Khadra M, Nanan R. A systematic review on incentive-driven mobile health technology: As used in diabetes management. J Telemed Telecare. 2017 Jan;23(1):26-35. PMID:26888421 |
| Desveaux L, Agarwal P, Shaw J, Hensel JM, Mukerji G, Onabajo N, Marani H, Jamieson T, Bhattacharyya O, Martin D, Mamdani M, Jeffs L, Wodchis WP, Ivers NM, Bhatia RS. A randomized wait-list control trial to evaluate the impact of a mobile application to improve self-management of individuals with type 2 diabetes: a study protocol. BMC Med Inform Decis Mak. 2016 Nov 15;16(1):144. PMID:27842539 |
| Georgsson M, Staggers N, Weir C. A Modified User-Oriented Heuristic Evaluation of a Mobile Health System for Diabetes Self-management Support. Comput Inform Nurs. 2016 Feb;34(2):77-84. PMID:26657618 |
| Goyal S, Morita P, Lewis GF, Yu C, Seto E, Cafazzo JA. The Systematic Design of a Behavioural Mobile Health Application for the Self-Management of Type 2 Diabetes. Can J Diabetes. 2016 Feb;40(1):95-104. PMID:26455762 |
| Greenwood DA, Gee PM, Fatkin KJ, Peeples M. A Systematic Review of Reviews Evaluating Technology-Enabled Diabetes Self-Management Education and Support. J Diabetes Sci Technol. 2017;11(5):1015-27. PMID:28560898 |
| Hilliard ME, Eshtehardi SS, Minard CG, Saber R, Thompson D, Karaviti LP, Rojas Y, Anderson BJ. Strengths-Based Behavioral Intervention for Parents of Adolescents With Type 1 Diabetes Using an mHealth App (Type 1 Doing Well): Protocol for a Pilot Randomized Controlled Trial. JMIR Res Protoc. 2018 Mar 13;7(3). PMID:29535081 |
| Hoppe CD, Cade JE, Carter M. An evaluation of diabetes targeted apps for Android smartphone in relation to behaviour change techniques. J Hum Nutr Diet. 2017 Jun;30(3):326-38. PMID:27747955 |
| Huang Z, Soljak M, Boehm BO, Car J. Clinical relevance of smartphone apps for diabetes management: A global overview. Diabetes Metab Res Rev. 2018;34(4):e2990. doi:10.1002/dmrr.2990 |
| Huckvale K, Adomaviciute S, Prieto JT, Leow MK-S, Car J. Smartphone apps for calculating insulin dose: a systematic assessment. BMC Med. 2015;13:106. PMID:25943590 |
| Issom DZ, Woldaregay AZ, Chomutare T, Bradway M, Årsand E, Hartvigsen G. Mobile applications for people with diabetes published between 2010 and 2015. Diabetes Manag. 2015;5(6):539-50 |
| Izahar S, Lean QY, Hameed MA, Murugiah MK, Patel RP, Al-Worafi YM, Wong TW, Ming LC. Content Analysis of Mobile Health Applications on Diabetes Mellitus. Front Endocrinol (Lausanne). 2017 Nov 27;8:318. PMID:29230195 |
| Karlsen B, Oftedal B, Lie SS, Rokne B, Peyrot M, Zoffmann V, Graue M. Assessment of a web-based Guided Self-Determination intervention for adults with type 2 diabetes in general practice: a study protocol. BMJ Open. 2016 Dec 13;6(12):e013026. PMID:27965253 |
| Weymann N, Harter M, Dirmaier J. Quality of online information on type 2 diabetes: a cross-sectional study. Health Promot Int. 2015 Dec;30(4):821-31. PMID:24688114 |
| Whittemore R, Zincavage RM, Jaser SS, Grey M, Coleman JL, Collett D, Delvy R, Ibrahim BB, Marceau LD. Development of an eHealth Program for Parents of Adolescents With Type 1 Diabetes. Diabetes Educ. 2018 Feb;44(1):72-82. PMID:29262747 |
| Ye Q, Khan U, Boren SA, Simoes EJ, Kim MS. An Analysis of Diabetes Mobile Applications Features Compared to AADE7TM: Addressing Self-Management Behaviors in People With Diabetes. J Diabetes Sci Technol. 2018 Jul;12(4):808-16. PMID:29390917 |
| **NO EVALUATION OR MEDICAL EVALUATION ONLY (n=2)** |
| Frandes M, Deiac AV, Timar B, Lungeanu D. Instrument for Assessing Mobile Technology Acceptability in Diabetes Self-management: A Validation and Reliability Study. Patient Prefer Adherence. 2017 Feb 14;11:259-69. PMID:28243069 |
| Hou C, Carter B, Hewitt J, Francisa T, Mayor S. Do Mobile Phone Applications Improve Glycemic Control (HbA1c) in the Self-management of Diabetes? A Systematic Review, Meta-analysis, and GRADE of 14 Randomized Trials. Diabetes Care. 2016 Nov;39(11):2089-95. PMID:27926892 |
| **NO SELF-MANAGEMENT APPS OR DIGITAL INTERVENTIONS (n=1)** |
| Lie SS, Karlsen B, Oord ER, Graue M, Oftedal B. Dropout From an eHealth Intervention for Adults With Type 2 Diabetes: A Qualitative Study. J Med Internet Res. 2017 May 30;19(5):e187. PMID:28559223 |
| **NO PRIMARY STUDIES (n=4)** |
| Fu HL, McMahon SK, Gross CR, Adam TJ, Wyman JF. Usability and Clinical Efficacy of Diabetes Mobile Applications for Adults with Type 2 Diabetes: A Systematic Review. Diabetes Res Clin Pract. 2017 Sep;131:70-81. PMID:28692830 |
| Kebede MM, Liedtke TP, Molleres T, Pischke CR. Characterizing Active Ingredients of eHealth Interventions Targeting Persons With Poorly Controlled Type 2 Diabetes Mellitus Using the Behavior Change Techniques Taxonomy: Scoping Review. J Med Internet Res. 2017 Oct 12;19(10):e348. PMID:29025693 |
| Moretti V, Morsello B. Self-management and Type 1 Diabetes: How Technology Redefines Illness. Tecnoscienza-Italian Journal of Science & Technology Studies. 2017;8(1):51-71 |
| Nelson LA, Coston TD, Cherrington AL, Osborn CY. Patterns of User Engagement with Mobile- and Web-Delivered Self-Care Interventions for Adults with T2DM: A Review of the Literature. Curr Diab Rep. 2016 Jul;16(7):66. PMID:27255269 |
