# Supplementary material for: Methods and Evaluation Criteria for Apps and Digital Interventions for Diabetes Self-Management: Systematic Review
Source: J Med Internet Res. 2020 Jul 6;22(7):e18480. doi: 10.2196/18480 (PMC7381260; doi:10.2196/18480)
Supplement: Multimedia Appendix 3 [file jmir_v22i7e18480_app3.doc]

**Multimedia Appendix 3**

Articles included in qualitative synthesis (n=31)

| **Reference** | **Evaluated platform and condition** | **Study population** | **Method of evaluation** | **Evaluation criteria** | **Quality assessment**  **(G-Grade; C-CerQual)** |
| --- | --- | --- | --- | --- | --- |
| [27] | Web for T2D | 48 individuals: Patients + HCPs | Focus group | Usability | High (C) |
| [33] | App for T1D | 63 Patients | Standardized questionnaires | Clinical impact; Cognitive impact | High (G) |
|  |  |  | Medical tests | Clinical impact |  |
|  |  |  | Self-reported health measures | Behavioral impact |  |
|  |  | 31 Patients | Standardized questionnaire | Usability |  |
| [36] | App for T1D | 20 Patients | Semi-structured interviews | Cognitive impact; Engagement | High (C) |
|  |  |  | Medical tests | Clinical impact |  |
|  |  |  | Standardized questionnaire | Cognitive impact |  |
| [43] | Web for T2D | 1041 Patients | Self-reported usage | Engagement | High (G) |
|  |  |  | Usage log analysis |  |  |
|  |  |  | Author-created questionnaire | Cognitive impact |  |
|  |  |  | Standardized questionnaire |  |  |
| [51] | Web for T2D | 1 Developer | Alpha testing | Usability | High (G) |
|  |  | 30 Patients | Other oral & written feedback |  |  |
|  |  | 59 Patients | Usage log analysis | Engagement |  |
|  |  |  | Author-created questionnaires | Usability; Acceptability and acceptance |  |
|  |  |  | Standardized questionnaire | Behavioral impact |  |
|  |  |  | Medical tests | Clinical impact |  |
| [52] | App for GDM | 17 Patients | Semi-structured interviews | Behavioral impact; Usability; Cognitive impact | High (G) |
| [54] | App for T2D | 75 Patients | Standardized questionnaires | Cognitive impact; Acceptability and acceptance | High (G) |
|  |  |  | Medical tests | Clinical impact |  |
|  |  |  | Usage log analysis | Engagement |  |
| [56] | App for T1D | 127 HCPs | Author-created questionnaires | Feasibility; Acceptability and acceptance | Moderate to high (C) |
|  |  | 245 Patients | Author-created questionnaires | Usability; Feasibility |  |
|  |  | 18 individuals: Patients + Informal Caregivers | Semi-structured interviews | Behavioral impact; Usability |  |
|  |  | Patients + Developers + Others | Workshop | Usability |  |
| [26] | Multiplatform for Unspecified diabetes type | 104 Patients | Anecdotal feedback | Acceptability and acceptance | Moderate (G) |
|  |  |  | Rating system | Usability |  |
|  |  | 8 individuals: Patients + Others | Workshop |  |  |
|  |  | 9 Others | Anecdotal feedback | Acceptability and acceptance |  |
|  |  |  | Rating system | Usability |  |
|  |  | 18 individuals: Patients + HCPs + Developers + Others | Workshop | Feasibility |  |
| [28] | Web for T1D & T2D | 21 Patients | Semi-structured interviews | Cognitive impact | Moderate (C) |
| [29] | App for T1D | 22 individuals: Patients + Informal Caregivers | Unspecified interview format | Usability | Moderate (C) |
|  |  | 24 HCPs | Unspecified interview format | Cognitive impact |  |
|  |  | Researchers + Others | Workshop | Security and privacy |  |
|  |  | 67 individuals: Patients + Informal Caregivers + HCPs + Others | Workshop | Usability |  |
|  |  | 49 individuals: Patients + Informal Caregivers + HCPs | Author-created questionnaire |  |  |
|  |  | 16 individuals: Patients + Informal Caregivers + HCPs | Think-aloud protocol |  |  |
|  |  | 6 Patients | Author-created questionnaire |  |  |
|  |  | 38 HCPs | Author-created questionnaire | Feasibility |  |
| [30] | App for T1D & T2D | 200 Patients | Author-created questionnaire | Usability | Moderate (G) |
| [31] | App for T2D | 16 Patients | Semi-structed interviews | Behavioral impact; Cognitive impact; Clinical impact | Moderate (C) |
|  |  |  | Medical tests | Clinical impact |  |
|  |  |  | Standardized questionnaire | Cognitive impact |  |
| [32] | App for T2D | 60 Patients | Semi-structured interviews | Usability | Moderate (G) |
|  |  | 15 Others | Author-created questionnaire |  |  |
| [35] | Web for GDM | 199 Patients | Usage log analysis | Feasibility | Moderate (G) |
|  |  | 73 Patients | Standardized questionnaire | Usability |  |
|  |  | 15 Patients | Semi-structured interviews | Behavioral impact; Usability; Feasibility |  |
| [37] | App for Unspecified diabetes type | 19 Patients | Focus group | Behavioral impact; Usability; Cognitive impact | Moderate (C) |
|  |  | 2 HCPs | Scenarios | Feasibility |  |
|  |  | 3 Others | Heuristics method | Usability |  |
|  |  | 14 Patients | Author-created questionnaires |  |  |
| [39] | App for Unspecified diabetes type | 90 Patients | Author-created questionnaires | Usability; Behavioral impact | Moderate (G) |
| [41] | App for T1D | 7 Patients | Focus group | Acceptability and acceptance; Usability | Moderate (C) |
| [42] | Multiplatform for T1D & T2D | 73 Patients + HCPs | Self-reported usage | Engagement | Moderate (G) |
|  |  |  | Self-reported health measures | Behavioral impact |  |
|  |  |  | Standardized questionnaires | Usability; Cognitive impact |  |
|  |  |  | Author-created questionnaires | Acceptability and acceptance; Behavioral impact |  |
|  |  | 9 Patients | Focus group | Behavioral impact |  |
| [45] | Web for GDM | 10 Patients | Semi-structured interviews | Acceptability and acceptance; Usability | Moderate (C) |
|  |  | 23 Patients | Self-reported health measures | Clinical impact |  |
|  |  |  | Medical tests |  |  |
|  |  |  | Standardized questionnaires | Cognitive impact; Behavioral impact |  |
|  |  |  | Usage log analysis | Feasibility |  |
|  |  | 10 Patients | Focus group | Feasibility; Usability |  |
| [46] | App for T2D | 28 Patients | Author-created questionnaires | Feasibility; Cognitive impact | Moderate (G) |
| [48] | App for T2D | 9 HCPs | Workshop | Usability | Moderate (C) |
|  |  | 19 Others | Observation |  |  |
|  |  | 12 Patients | Semi-structured interviews |  |  |
|  |  | 14 Patients | Semi-structured interviews | Feasibility; Engagement |  |
|  |  | 7 HCPs | Workshop | Feasibility |  |
| [49] | App for T2D | 11 Patients | Semi-structured interviews | Behavioral impact; Cognitive impact | Moderate (C) |
|  |  |  | Medical tests | Clinical impact |  |
| [53] | Web for Unspecified diabetes type | 16 Patients + Informal Caregivers | Semi-structured interviews | Feasibility; Usability; Engagement | Moderate (C) |
| [55] | Web for Unspecified diabetes type | Patients | Author-created questionnaires | Acceptability and acceptance; Usability | Moderate (G) |
|  |  | Patients | Anecdotal feedback | Usability |  |
|  |  | Patients | Medical tests | Clinical impact |  |
|  |  | Others | Cost-effectiveness | Feasibility |  |
|  |  | 175 Patients | Author-created questionnaire |  |  |
|  |  | 279 Patients | Author-created questionnaire | Usability |  |
| [34] | App for T2D | 10 Patients | Usage log analysis | Usability | Low (G) |
|  |  |  | Standardized questionnaire |  |  |
| [38] | App for GDM | 6 individuals: Others | Author-created questionnaire | Feasibility | Low (G) |
|  |  | 3 Researchers + Others | Scenarios |  |  |
|  |  | Patients | Author-created questionnaire | Acceptability and acceptance |  |
|  |  | 22 Patients |  |  |  |
|  |  | 5 Patients | Unspecified interview format | Usability |  |
|  |  |  | Standardized questionnaire |  |  |
| [40] | Multiplatform for T1D | 7 Patients | Focus group | Usability | Low (C) |
|  |  |  | Standardized questionnaires | Cognitive impact; Usability |  |
|  |  | 14 Patients |  |  |  |
|  |  |  | Focus group | Usability |  |
| [44] | Multiplatform for T1D | 32 Patients + Informal Caregivers | Other oral & written feedback | Usability | Low (C) |
|  |  | Researchers + Developers | Security assessment | Security and privacy |  |
| [47] | App for T2D | 18 Patients | Focus group | Acceptability and acceptance; Feasibility; Engagement | Low (C) |
| [50] | Web for T2D | 7 Patients | Standardized questionnaires | Cognitive impact; Clinical impact | Low (G) |
